# Choosing a stable housekeeping gene and protein is essential in generating valid gene and protein expression results

**DOI:** 10.1038/bjc.2011.35

**Published:** 2011-03-01

**Authors:** M Sullivan-Gunn, E Hinch, V Vaughan, P Lewandowski

**Affiliations:** 1 Molecular Nutrition Unit, School of Medicine, Deakin University, Waurn Ponds 3217, Australia

**Sir,**

We have read and support the concerns of Caradec *et al* (2010) in the *British Journal of Cancer* regarding the stability of GAPDH as an internal control gene that was used in the recent study by Kontos *et al* (2010) to normalise L-DOPA decarboxylase mRNA as a prognostic marker in colorectal adenocarcinoma. We too have recently found variance in protein and gene expression of the commonly used housekeeping genes, *GAPDH* and ribosomal 18s, in the hearts of colon adenocarcinoma-bearing mice. Although differences in GAPDH mRNA were not statistically significant, results show some variation in GAPDH protein levels between murine adenocarcinoma (MAC) models of tumour-bearing cachectic (MAC16) and non-cachectic mice (MAC13; *P*=0.05). We have also found significant variation in the gene expression of ribosomal 18s in the hearts of these two MAC cancer models (*P*<0.001) and in each cancer model compared with non-tumour-bearing control mice (*P*=0.004; *P*<0.001). Further to this, variations in ribosomal 18s expression were observed at different stages of cancer development in the hearts of MAC13 tumour-bearing mice compared with control mice. Ribosomal 18s gene expression in the hearts of MAC13 tumour-bearing mice showed significant differences at days 12 (*P*=0.011) and 21 (*P*=0.047), when compared with day 29 after implantation.

Although still widely used as housekeeping genes, *GAPDH* and ribosomal 18s expression levels have been shown to vary under different experimental conditions (Mahoney *et al*, 2004; De Santis *et al*, 2010; Nelissen *et al*, 2010), and disease states (Mahoney *et al*, 2004; De Santis *et al*, 2010; Nelissen *et al*, 2010) showed GAPDH to be highly variable in cancerous compared with non-cancerous tissue. Nelissen *et al* (2010) recently described GAPDH and ribosomal 18s as the most unstable housekeeping genes under different experimental conditions in qPCR experiments with rat oligodendrocytes. The use of variable housekeeping genes as normalisers will lead to misinterpretation and irrelevant results.

Thoughtful consideration as well as evaluation of a valid housekeeping gene that is ubiquitously expressed and stable across sample groups is absolutely necessary to obtain reliable gene expression results and eliminate misinterpretations. Alternatively, fluorescein-labeled oligonucleotides such as OliGreen (Invitrogen, Mulgrave, Victoria, Australia), which measures cDNA content in each sample, may be a more reliable reference for normalising protein and gene expression results (Lundby *et al*, 2005; Rhinn *et al*, 2008).

## References

[bib1] Caradec J, Sirab N, Revaud D, Keumeugni C, Loric S (2010) Is GAPDH a relevant housekeeping gene for normalisation in colorectal cancer experiments? Br J Cancer 103: 1475–1476 20859286 10.1038/sj.bjc.6605851 PMC2990595

[bib2] De Santis C, Smith-Keune C, Jerry D (2010) Normalizing RT-qPCR data: are we getting the right answers? An appraisal of normalization approaches and internal reference genes from a case study in the Finfish Lates calcarifer. Mar Biotechnol (NY) (e-pub ahead of print 23 March 2010; doi:10.1007/s10126-010-9277-z) 10.1007/s10126-010-9277-z 20309600

[bib3] Kontos C, Papadopoulos I, Fragoulis E, Scorilas A (2010) Quantitative expression analysis and prognostic significance of L-DOPA decarboxylase in colorectal adenocarcinoma. Br J Cancer 102: 1384–1390 20424616 10.1038/sj.bjc.6605654 PMC2865762

[bib4] Lundby C, Nordsborg N, Kusuhara K, Kristensen K, Neufer P, Pilegaard H (2005) Gene expression in human skeletal muscle: alternative normalization method and effect of repeated biopsies. Eur J Appl Physiol 95: 351–360 16151837 10.1007/s00421-005-0022-7

[bib5] Mahoney D, Carey K, Fu M, Snow R, Cameron-Smith D, Parise G, Tarnopolsky M (2004) Real-time RT-PCR analysis of housekeeping genes in human skeletal muscle following acute exercise. Physiol Genomics 18: 226–231 15161965 10.1152/physiolgenomics.00067.2004

[bib6] Nelissen K, Smeets K, Mulder M, Hendriks J, Ameloot M (2010) Selection of reference genes for gene expression studies in rat oligodendrocytes using quantitative real time PCR. J Neurosci Methods 187: 78–83 20036692 10.1016/j.jneumeth.2009.12.018

[bib7] Rhinn H, Scherman D, Escriou V (2008) One-step quantification of single-stranded DNA in the presence of RNA using Oligreen in a real-time polymerase chain reaction thermocycler. Anal Biochem 372: 116–118 17963709 10.1016/j.ab.2007.08.023

